# The National Evaluation Platform for Maternal, Newborn, and Child Health, and Nutrition: From idea to implementation

**DOI:** 10.7189/jogh.07.020305

**Published:** 2017-12

**Authors:** Rebecca Heidkamp, NEP Working Group

**Affiliations:** Institute for International Programs, Johns Hopkins Bloomberg School of Public Health, Baltimore, Maryland, USA; * Membership of the NEP Working Group is listed in the Acknowledgments

Accelerating progress in women’s and children’s health requires scaling up efficacious interventions and measuring progress towards defined targets. However, determining what is effective in a particular setting and optimizing investments is challenging given the complexity of health systems and the diversity of contexts surrounding maternal, newborn, and child health and nutrition (MNCH&N) policies and programs in low– and middle–income countries (LMICs). There have been various global efforts to synthesize evidence (eg, World Health Organization Guidelines; various Lancet series on maternal child health and nutrition issues, Cochrane Collaborative reviews, Disease Control Priorities Project and monitor progress towards shared goals (eg, Sustainable Development Goals, World Health Assembly 2025 Nutrition Targets, the Countdown to 2030, Family Planning 2020) which have some influence on country-level priorities and plans [1–6]. Ultimately, however, national and sub-national stakeholders want evidence from their country to guide their policy and program decisions. Too often this evidence is not available when and where decisions makers need it.

Photo: Two task team members in Tanzania. From the private collection of Rebecca Heidkamp.

The National Evaluation Platform (NEP) is a systematic approach to identifying, compiling, and rigorously analyzing data from diverse sources (eg, household and facility surveys; administrative data), in order to evaluate the effectiveness of MNCH&N policies and programs. Country–led and country–owned, the NEP approach offers a set of core evaluation methods and tools to build national capacity for generating evidence–based answers to program and policy questions. NEP complements and reinforces other ongoing efforts to strengthen country monitoring and evaluation (M&E) systems and to promote data use.

We present the history and rationale underlying NEP, describe core components and work streams supporting NEP implementation in four African countries, and introduce a collection of peer–reviewed articles about NEP to be published in the *Journal of Global Health* over the coming year.

## HISTORY AND RATIONALE FOR NEP

The NEP concept took root during an evaluation of the scale–up of Integrated Community Case Management (iCCM) programs in Malawi conducted by the National Statistics Office (NSO) and the Institute for International Programs at the Johns Hopkins Bloomberg School of Public Health (IIP–JHU) with financial support from the Government of Canada [7]. The original evaluation plan was developed in late 2008 using a quasi–experimental pre–post design that compared outcomes in six intervention districts where UNICEF and WHO were supporting iCCM implementation to six comparison districts where no iCCM activities were planned. However, by the end of 2009, iCCM had been scaled–up to all of 28 districts in Malawi with technical and financial support from other partners. With the original comparison districts no longer able to serve in this capacity, the NSO and IIP–JHU evaluation team proposed a “dose–response” design that included all 28 districts in Malawi and aimed to assess whether districts with stronger iCCM implementation had stronger impact. The dose (implementation strength) was measured through newly collected routine and survey data while the response relied on measures of outcome (treatment and intervention coverage) and impact (child mortality) from nationally–representative household surveys that were not specific to the evaluation. The evaluation team coined this approach – using a dose–response analysis with data from a combination of sources a *NEP* design. Findings were published in 2016 [8].

In the wake of the Malawi iCCM evaluation redesign, a group of experts in MNCH&N program evaluation published an article in the Lancet that proposed NEP as a departure from the status quo in large–scale effectiveness evaluations [9]. Rigorous evaluations of real–world programs at scale are rare, and those that do happen often focus on a single program area such as HIV or malaria rather than the full MNCH&N continuum of care delivered at health systems and community levels. They tend, like the original Malawi iCCM evaluation, to rely on intervention vs control designs that assume there are comparison areas where no related MNCH&N programs exist and where conditions will be relatively constant across the evaluation period. They typically focus on select subnational areas of interest to specific donors – limiting their utility to national governments who need to make decisions for their entire population. Finally, these approaches can be costly as they often require new data collection across multiple time points [8].

In contrast, the NEP approach articulated by Victora et al. aims to answer evaluation questions that are formulated based on MNCH&N program impact pathways developed using a common framework formulated by Bryce and others [8,9]. Data characterizing the inputs, processes, outputs, outcomes, impacts, and contexts of MNCH&N interventions and programs are assembled to the fullest extent possible from existing survey and routine data sources. The quality of data are assessed and then they are organized by district in a way that facilitates co–analysis and can be expanded as new data become available. NEP analyses are observational. They examine differences across districts (or other sub–national units) for key indicators along the impact pathways using time trends, equity, and regression methods as well as the Lives Saved Tool. NEP is not intended to replace intervention efficacy trials nor does it preclude the need for other types of evaluations. Rather it aims to address the practical needs of LMIC stakeholders for timely evidence to drive high–level MNCH&N policy and program decision making.

## IMPLEMENTATION OF NEP FOR MNCH&N

In late 2013, IIP–JHU received funding from Global Affairs Canada (GAC) to take NEP from a concept applied in a single evaluation in Malawi to a sustainable approach to rigorous evaluation by public sector MNCH&N stakeholders in four sub–Saharan African countries–Malawi, Mali, Mozambique and Tanzania. Country–level roll–out began in early 2014. By December 2017, IIP–JHU and government partners in each country aim to build systems and institutional capacity to carry out analyses and communicate NEP findings and to demonstrate that NEP outputs influence decision making by MNCH&N policy and program stakeholders. Activities are organized under three work streams which we highlight below: country–level operationalization; core technical development; and documentation, evaluation and communication.

Public sector institutions lead every aspect of NEP implementation. NEP brings together key government institutions involved in MNCH&N program evaluation, including those that make policies, implement programs, collect and report data, conduct research, and/or oversee budgets. Depending on the country this may include several units under the ministry of health, national statistical offices, multi–sectorial nutrition coordination bodies, ministries responsible for finance and local administration, public universities, and/or public health research institutes. Each country has a designated *NEP Home Institution* with several staff dedicated to NEP oversight and implementation, including data management and stakeholder coordination. A country–specific *NEP High–level Advisory or Steering Committee* is made up of senior leaders from MNCH&N stakeholder institutions in and outside government who guide NEP implementation by recommending or endorsing priority questions and serving as the first audience for NEP findings. The *NEP Technical Task Team* includes staff from each of the country’s NEP stakeholder institutions who work in M&E, program coordination or data analytics. The Task Team is the “engine” of NEP, with members working together to develop core evaluation skills, answer evaluation questions and ensure that their respective institutions support and utilize NEP. IIP–JHU has one full–time staff member in each country who coordinates technical assistance, capacity building, and networking. A diverse team of faculty based at Johns Hopkins University (Baltimore, USA) support technical development and implementation along with several external partners including Health Alliance International (Seattle, USA) and 2Paths (Vancouver, Canada).

**Figure 1:**
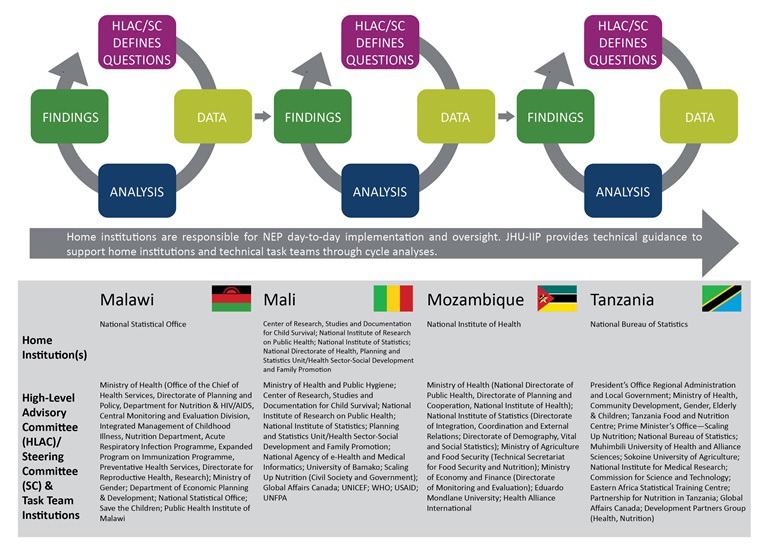
National Evaluation Platform (NEP) structure.

Country teams adopt a “cycle–based” approach to establishing core NEP systems and building capacity to carry out the evaluation work (**Figure 1**). A cycle is driven by specific evaluation questions identified and prioritized by local MNCH&N stakeholders. Each cycle of NEP development progressively adds new types of data, new analytical skills and new communications approaches to disseminate findings to policy maker and program planner audiences. IIP–JHU has adapted or developed a set of flexible tools to support each step in the NEP cycle including question development, data quality assessment, data management, statistical analysis and communications. For example, the innovative *Stats Report* tool helps address limitations in statistical expertise and capacity to use statistical software by allowing users to select, adapt and run core analysis, data management and data quality assessment functions programmed in R using a simple interface. IIP–JHU provides targeted mentorship and curriculum tailored to each country team’s existing capacity. The overall NEP curriculum is organized by eight core technical areas that comprise modules to introduce and apply relevant skills. These technical areas include: (1) general evaluation principles; (2) core data concepts; (3) data mapping; (4) data quality assessment; (5) data management; (6) data analysis; (7) new data collection; and (8) interpretation & reporting. Learning modules are designed to be customized and used by other groups wanting to adopt NEP methods.

The effectiveness of NEP will be judged by the extent to which the evidence produced by country teams is incorporated into decision–making processes for women and children’s health and nutrition. There are promising signs of NEP influence across the four countries including in Mali where the first cycle resulted in a call by MOH leadership to harmonize maternal child health plans and targets as well as in Tanzania where cycle 1 results were used to develop the next health sector strategic plan. Overall progress across the four countries is being evaluated by an external partner, FSG Social Impact, and findings are used to improve ongoing NEP roll–out and to arrive at summary judgments of NEP effectiveness. These efforts are complemented by extensive internal documentation by IIP–JHU of the planning, decision making, and implementation process during the four years.

## AIMS OF THE COLLECTION

Over the coming year, the *Journal of Global Health* will publish a series of peer–reviewed papers related to NEP. Together the articles in the collection will: 1) describe and demonstrate core NEP design features including innovative methods and tools supporting data quality assessment, data management, data analysis, and capacity building; 2) present analyses produced by NEP country teams in response to locally–prioritized evaluation questions and identify how findings have been used by national MNCH&N stakeholders, and 3) assess whether the project has met high–level objectives including the potential for NEP to be sustained in current countries and successfully replicated and refined by other countries or sectors. Ultimately, we believe that sharing our outputs and overall experience in implementing NEP will encourage dialogue among academics, donors, and policymakers on the need to support governments in using data and developing evidence that guides their MNCH&N decision making.
